# Psychobiographical Reflections on Marilyn Yalom’s Experience of Death and Dying

**DOI:** 10.5964/ejop.19089

**Published:** 2025-11-28

**Authors:** Carla Nel

**Affiliations:** 1 Department of Psychology, University of the Free State, Bloemfontein, South Africa; University of Johannesburg, Johannesburg, South Africa

**Keywords:** Marilyn Yalom, Irvin Yalom, existential concerns, mortality, dying, end-of-life, terminal illness

## Abstract

The universal awareness of death is central to the framework of existential psychology. However, its subjective confrontation remains deeply personal. Marilyn Yalom (1938–2019), distinguished feminist scholar and cultural historian, documented her intimate reflections on dying in a book co-authored with her husband, Irvin Yalom, renowned existential psychotherapist and author. This single-case psychobiography sought to examine her experience, focusing on her diarised reflections on living with and dying from a terminal illness in this book as the primary data source. Data analysis examined Marilyn’s reflections through the lens of Irving Yalom’s existential psychotherapy propositions to construct a theoretically informed overview of her experiences. Given her observation of feeling prepared to face death as a concept, as opposed to dying as a process, thematic analysis uncovered additional insights regarding this transition. Traditionally, psychobiographies explore the lives of significant figures in their entirety, and few have focused specifically on an individual’s confrontation with death. The findings are presented within the framework of the four existential concerns, and additional experiential themes are conceptualised as end-of-life variations of lifelong existential concerns. These illuminated underexplored concerns, such as the process of detaching from loved ones, physician-assisted suicide as an expression of autonomy, and the potential for pain and impairment to re-awaken the crises of isolation and meaninglessness. Practice recommendations are made from the findings, in line with Marilyn and Irving Yalom’s goal of contributing to a broader discourse on end-of-life concerns, as Marilyn endeavoured to fight against despair and live meaningfully until the very end.

An awareness of death as a universal human experience was central to developing existential approaches to psychology (Feifel, 1969; May, 1969; Yalom, 1980). Emerging from the thinking of a diverse range of European philosophers and writers, Existentialism was incorporated into psychotherapy frameworks throughout the 20^th^ century (Frank, 2022; Kőváry, 2024; Lindström et al., 2025). Existential analysts, such as Binswanger and Frankl, as well as existential humanistic psychologists, such as Murray, Maslow and Rogers, developed existential thought within different psychological paradigms, all with the central emphasis on the profound anxieties that emerge when individuals confront the finitude and freedom of existence, resulting in a universal quest for meaning (Berman, 2019; Frank, 2022; Kőváry, 2024).

Irvin Yalom, whose work was deeply influenced by Rollo May and American psychoanalysis, became a popular existential-humanistic figure outside the boundaries of formal existentialist schools or movements (Kőváry, 2024). He widely popularised the proposition that humans are inevitably confronted with four existential concerns with which they must actively engage (Binder, 2022; Kőváry, 2024). In Yalom’s existential psychotherapy (Yalom, 1980), *death*, as the first concern, represents an awareness of the inevitability of the end of life, which evokes anxiety that can either paralyse or drive individuation and authenticity. The second concern, *freedom*, refers to the responsibility of authorship over one’s life in the face of seemingly limitless possibilities through ownership of one’s choices and embracing one’s capacity for self-determination. The third concern, *isolation*, refers to the fundamental unbridgeable gap between individuals, even within the most intimate relationships, resulting in the need to build meaningful connections within the limits of existential isolation. Lastly, *meaninglessness* refers to the absence of an inherent purpose in life, compelling individuals to construct a sense of significance, lest they experience an existential vacuum (Yalom, 1980).

Despite the universality of death on an abstract, philosophical level, the subjective confrontation with one’s own death remains deeply personal. As a biological process and a personal event, dying did not receive much attention within existentialist writings (Peach, 2000). The medicalisation of human distress (Binder, 2022), as well as the biomedical lens through which the over-85 age group is often viewed (van Rhyn et al., 2022), has resulted in a distinct dearth of research among those who grapple most intimately with their mortality. However, the exploration of patients’ existential reflections has received renewed interest within broader health research (Lindström et al., 2025).

Professor Marilyn Yalom (1938–2019), a distinguished feminist scholar and cultural historian (Berman, 2019; Reisz, 2020), documented her intimate reflections on dying in *A Matter of Death and Life: Love, Loss and What Matters in the End*, co-authored with her husband, Irvin Yalom (Yalom & Yalom, 2021). In the preface, Marilyn highlighted specific questions she faced after her multiple myeloma diagnosis, namely, how to fight against despair and how to live meaningfully until the very end (Yalom & Yalom, 2021). Traditionally, psychobiographical studies explore the lives of significant figures in their entirety (Mayer et al., 2021; Ponterotto, 2025). A specific focus on an individual’s confrontation with death and the navigation of these end-of-life questions presents a significant gap in current psychobiographical research, prompting this study to undertake an existentially informed analysis of Marilyn Yalom’s end-of-life reflections.

## Research Aim and Methodology

The primary aim of this study is to examine and explore the subject’s narrative of her personal experience of dying within the framework of Yalom’s four existential concerns. As a secondary aim, this study undertook to explore any additional narrative themes related to her confrontation with her own imminent death. To meet these aims, this qualitative single case study followed a psychobiographical design. Psychobiography, as a subdivision of psychohistory, allows for a deeper understanding of a public figure’s inner world within their socio-historical context by reconstructing a subject's inner experiences within the framework of a psychological theory or model (Anderson, 2024; Ponterotto, 2025) and their socio-cultural context (Mayer & van Niekerk, 2024) within the constructivist-interpretivist paradigm (Fouché et al., 2014; Fouché et al., 2018). Psychobiographical inquiries into meaning-making have recently surfaced, drawing on diverse frameworks, including existential theory (Mayer et al., 2021). The continued use of existential theory in psychobiography, such as by Harry and van Niekerk (2023), has supported the applicability of the psychobiographical approach to the exploration of existential themes.

### Participants and Sampling

Purposive sampling allows psychobiographers to select a subject to best meet their objectives (van Niekerk et al., 2019). Marilyn Yalom (1938–2019) was accordingly selected because of her production and publication of a detailed narrative account of her last few months of life. She described her experiences openly, clearly, and with insight into existential themes, rendering her an ideal subject to meet the research aim.

### Data Collection, Extraction and Analysis

Data collection focused primarily on her reflections published in *A Matter of Death and Life: Love, Loss and What Matters in the End* (Yalom & Yalom, 2021), as a data source, as it contains her narrative accounts on being terminally ill, in the form of monthly diary entries. Additional publicly available information on Marilyn’s life and work was consulted to contextualise and interpret her reflections.

Data was extracted using Alexander’s (1988, 1990) two proposed methods for psychobiographical data extraction, namely, (a) questioning the data by searching for instances of how the propositions of the theoretical framework present themselves in the narrative; and (b) extracting additional psychologically salient data for further analysis. First, Marilyn’s reflections were extensively reviewed, and excerpts from the text were coded based on the four existential concerns highlighted by Irvin Yalom (1980). This aligns with Alexander’s (1988, 1990) first strategy of “asking the data questions”. These questions are aimed at finding revelatory statements about the subject’s acceptance of, (a) her own mortality, (b) her responsibility and agency in the face of freedom, (c) her existential isolation in the context of meaningful connection, and (d) her role in creating meaning in her life. Second, Alexander’s (1988, 1990) proposed saliency indicators were used to identify additional psychologically relevant information from biographical data. These indicators (frequency, emphasis, uniqueness, primacy, repetition, interruption, isolation, incompletion and distortion) help researchers organise and prioritise information, highlighting elements of unusual prominence and psychological importance (Alexander, 1988, 1990).

The psychobiographical approach entails the application of the propositions of the selected theoretical framework to the biographical data (Ponterotto, 2014, 2025). Data analysis, therefore, examined Marilyn’s reflections through an existential psychology lens to construct a theoretically informed overview of her experiences. Given the subject’s narrative reflection of how she felt prepared to face death as a concept, as opposed to dying as a process, thematic analysis (Braun & Clarke, 2012) was also used to examine the additional psychologically salient data from Marilyn’s narrative accounts, to uncover any additional themes that might fall outside the propositions of the existential concerns. These psychologically salient statements were coded and subsequently clustered into themes. Therefore, deductive and inductive processes of inquiry were utilised to fully explore one individual’s experience of facing death.

### Ethical Considerations

The proposal was submitted for ethical review to the General/Human Research Ethics Committee of the University of the Free State (Reference Number: UFS-HSD2025/0548). Ethical principles for psychobiographical research proposed by Ponterotto (2014, 2025) informed the design of this study. Therefore, the sampled subject is deceased, and only publicly available information was included in the collection and analysis of data to protect living relatives from previously unknown information on the subject’s life.

## Findings and Discussion

Marilyn Koenick was born in Chicago in 1932 and raised in Washington (Parker, 2019; Reisz, 2020). Her relationship with Irvin Yalom started when she was fourteen, as they bonded over their love of books (Berman, 2019). After she obtained a diploma from the Sorbonne and graduated from Wellesley College with a BA degree in French, the couple married in 1954 (Berman, 2019). Marilyn obtained her master’s degree from Harvard University in 1956 and completed her PhD in Comparative Literature at Johns Hopkins University in 1963, in which she focused on the writings of Kafka and Camus. She joined Stanford University in 1976, serving as senior scholar at the Michelle Clayman Institute for Gender Research, where she played a pivotal role in establishing the feminist studies programme (Berman, 2019; Parker, 2019). Marilyn, an admirer of 18^th^-century French salon culture, took the lead in organising events of intellectual discourse and hosted regular literary salons for fellow women scholars (Clayton, 2020; Parker, 2019). She authored numerous influential books, including *Blood Sisters* (1993), *A History of the Breast* (1997), *A History of the Wife* (2001), *Birth of the Chess Queen* (2004), *How the French Invented Love* (2012), *The Social Sex: A History of Female Friendship* (2015), *Compelled to Witness: Women's Memoirs of the French Revolution* (2015) and *The Amorous Heart: An Unconventional History of Love* (2018). She collaborated with her son, Reid, on *The American Resting Place: Four Hundred Years of History Through Our Cemeteries and Burial Grounds* (2008). Her work used a transnational and historical lens to explore themes such as the representations of women in history, marriage, love, and friendship (Clayton, 2020; Parker, 2019; Reisz, 2020).

Throughout their six decades of marriage, Marilyn was also closely involved with her husband’s writings, and they acted as each other’s first reader and editor (Yalom & Yalom, 2021). The couple travelled extensively and enjoyed regular contact with their four children and eight grandchildren (Berman, 2019; Kenrick, 2021; Yalom & Yalom, 2021). Marilyn received multiple honours, such as being named *Officier des Palmes Académiques* by the French government in 1992 and receiving Wellesley College’s Alumnae Achievement Award (Parker, 2019; Reisz, 2020). A gifted communicator, she was lauded for fostering intellectual community, especially among women scholars, and she remained an influential voice in feminist cultural history until her death (Clayton, 2020; Parker, 2019).

When chemotherapy and immunoglobulin therapy proved unsuccessful in treating her multiple myeloma, Marilyn opted for physician-assisted suicide on 20 November 2019 in the presence of Irving, their children, her physician and her nurse (Kenrick, 2021; Yalom & Yalom, 2021). That year, she was one of 405 people who accessed this option under Californian legislation (Kenrick, 2021). Her son, Ben, said: “We were lucky to be loved and mentored by our mother, and to share her with so many wonderful people... She had a wonderful life, lived without regrets. Dying well, she continued to teach us.” (Parker, 2019, Par. 23).

The results from the data analysis on the presence of the four existential concerns in her published reflections are presented and discussed in the following sections. Marilyn’s reflections offer a deeply personal account of coming to terms with the inevitability of death, richly coloured by her experiences of advanced age and terminal illness. Salient statements also revealed additional end-of-life variations to these lifelong existential concerns. These represent experiences in which the four existential crises are renewed and embodied anew in the context of dying. Therefore, these themes will be presented and discussed within the framework of existential concerns below.

### Existential Concern 1: Death

Throughout the text, Marilyn wrote of death with a tone of rational acceptance, as she acknowledged, “I am 87 years old... a ripe time to die” (Yalom & Yalom, 2021, p. 13) and noted that she has already enjoyed “a very satisfying long life” (p. 13), surpassing the average life expectancy. With this pragmatic acknowledgement, she also doubted the value of prolonging her life in the face of “daily misery and despair” (Yalom & Yalom, 2021, p. 13). Early in the text, her acceptance of the timing of her death was tempered by a subtle undercurrent of disappointment in not matching her mother’s longevity, who “died peacefully at the age of 92 ½. I always assumed that I would die at her age...” (Yalom & Yalom, 2021, p. 18). She also reflected on gendered expectations of mortality, noting, “How odd that I should be the one who will probably die first, when statistically it is more frequently the husband who dies first” (Yalom & Yalom, 2021, p. 63). Her linguistic analysis of the term “widower” as a derivative of “widow” underscored this expectation's cultural and grammatical encoding.

Marilyn did not shy away from expressing the emotional complexity of her experience, articulating feelings of sadness and dread in the face of uncertainty, particularly regarding the potentially painful physical experiences of dying. Drawing on a friend’s distinction between *la mort* (death as a state) and *mourir* (the act of dying), she revealed a fear not of death itself, but of the suffering that may precede it. She noted:

“I, too, am not afraid of death itself, but the process of dying in daily doses is often intolerable… I have been accustoming myself to the idea of my upcoming death. Since Irv and I have contemplated the subject of death for decades, … I seem able to confront the idea with a degree of calm that surprises my friends. Sometimes I wonder if the calm is only a veneer and that underneath, I, too, am terrified.” (Yalom & Yalom, 2021, p. 65)

She proceeded to provide an example of how her “well of hidden anguish” (Yalom & Yalom, 2021, p. 66) in response to her own decline found expression in a vivid dream. Marylin described that in the dream, she was talking to a friend on the phone, who informed her that her adult son had died. She recalled: “I start to scream and awake convulsing with tears. In real life, that friend does not even have a son. So, whose death am I crying about? Probably my own” (p. 66).

She also reflected on moments that provide comfort and promote an acceptance of death, reached mainly through active connection and contemplation. For example, she noted finding solace in the care and concern received from friends and loved ones, including the prayers of friends from diverse religious backgrounds, even though she does not believe in consciousness after death. She noted that religious and literary references now resonated with new significance for her: “familiar poetic phrases take on new meaning in my present situation, as I lie on the sofa and reflect” (Yalom & Yalom, 2021, p. 16). For example, when uncertainties arose about whether she would still be alive for a specific future event, she noted: “I fall back on the words from Ecclesiastes: ‘There is a time for every season... A time to be born and a time to die’” (Yalom & Yalom, 2021, p. 49).

She began to describe death not only as inevitable, but as merciful: “…palliative care and assisted suicide would be a relief if my present treatment is not effective” (Yalom & Yalom, 2021, p. 64). Later in the text, her reflections reveal a more resolute acceptance of death’s imminence as she recounts a dream that signals a definitive shift from philosophical acceptance to readiness. This dream, she noted, “… doesn’t take my psychiatrist husband long to analyze… and see in it my desire to end an agonizing life” (Yalom & Yalom, 2021, p. 75). This shift seems to be supported by the regaining of some autonomy over her final moments once she and her hospice care team had discussed the option of physician-assisted suicide: “In the end, however that comes, I should have some measure of control” (Yalom & Yalom, 2021, p. 123).

However, against the backdrop of increased calm in her acceptance of death, she described how her emotional experience remains coloured by “continued sadness” (Yalom & Yalom, 2021, p. 123), stemming from the anticipation of separating from loved ones: “For all the philosophical treatises and for all the assurances of the medical profession, there is no cure for the simple fact that we must leave each other” (p. 123). This sadness emerged from Marilyn’s reflections as a prominent end-of-life theme related to the existential concern of mortality, namely, the relational considerations in the process of accepting death. This theme highlights how the mourning of one’s own demise is often tied to the anticipated disconnection from loved ones, further complicated by concerns over *their* grief. This idea echoed throughout her entries, as she noted in a later chapter (wittingly titled *Whose death is this anyway?)*: “It occurs to me… that my death is not mine alone. I shall have to share it with those who love me” (Yalom & Yalom, 2021, p. 64). Marilyn admitted “…it is difficult to accept that I will not be around to watch my three youngest grandchildren grow,” and added “they will not know me, except in flitting memories” (Yalom & Yalom, 2021, p. 43). She later added that she “cannot think ahead to Irv’s widowerhood. It saddens me greatly to imagine him alone” (Yalom & Yalom, 2021, p. 63) and described being “moved and unsettled” (p. 63) by her husband’s anticipatory grief. Death, therefore, is seen as particularly difficult to accept when it implies non-existence in the lives and memories of loved ones, and when it results in their pain.

### Existential Concern 2: Freedom

Marilyn’s reflections throughout the text reveal a lifelong recognition of personal agency and indicate that she valued self-determination. She recounted achievements, milestones and examples of living according to her valued principles (Yalom & Yalom, 2021). She also reflected on how her husband’s writing about the unlived life has been influential in helping her accept death, stating: “I am one of the lucky ones who will die with no regrets” (Yalom & Yalom, 2021, p. 77). Marilyn also set clear intentions for her remaining time, stating her desire to “rise to my best self and express my love… and, with grace, accept my fate” (Yalom & Yalom, 2021, p. 121). She later revisited the idea of how acceptance of one’s degree of satisfaction with the choices throughout one’s life also affects one’s acceptance of death:

“…I do believe that dying persons – when they have time to reflect – tend to evaluate the lives they have lived. Certainly, that is my case. And without being self-satisfied in a negative sense, I feel that I have caused no harm and can come to my end with few regrets and little guilt. The many emails, cards and letters I have received keep telling me that I was helpful in significant ways to a number of people. That is certainly one of the reasons that I feel calm most of the time and can anticipate the possibility of a ‘good death’.” (p. 122)

Her reflections illuminate the many limitations imposed by ageing, illness, and imminent death as she described the remaining options in her final months of life. For example, she reflected on the choice between assisted living and remaining in her home. While she acknowledged the conveniences and benefits of institutional “round-the-clock care” with “meals prepared and served” (Yalom & Yalom, 2021, p. 29), she also appreciated the impact of her attachment to her home: “It is comforting to move among familiar objects for the last period of our life” (p. 34). She reflected on the privileges that enable her choice, such as the financial means to make modifications to their home for her decreased mobility and to maintain the employment of their long-term housekeeper (Yalom & Yalom, 2021). Her reflections also reveal the difficulty of such decisions: “We sometimes ask ourselves: Are we making a mistake…? But the thought of leaving our home of forty-plus years … deters us. We are simply not willing to give up this house and setting…” (Yalom & Yalom, 2021, p. 29). This also suggests that personal agency may not, in the context of such a lifelong marriage, be easily unravelled from shared decision-making.

In the face of the illness-related limitations towards the end of her life, she noted her commitment to her remaining choices, which revolve around how to continue living according to her lifelong values of kindness. She reflected on the intentional selection of examples worthy of emulation, referring to a friend as “my role model of how I would like to behave in the months to come” as she recalled her grace and loving kindness, adding: “I noticed how respectfully she addressed the nurses as they came in and out of the room” (Yalom & Yalom, 2021, p. 18). She wrote: “I carry within me the memory of parents and teachers and colleagues, who were generous and loving,” and concluded that “now, as my time on earth draws to a close, I am trying to live out my remaining days in accordance with those principles” (Yalom & Yalom, 2021, p. 47). This includes choices about taking leave of friends: “I do not want to disappear from their lives without letting them know how much they have meant to me” (Yalom & Yalom, 2021, p. 103).

Marilyn’s refusal to pursue more aggressive medical interventions, such as stem cell or bone marrow transplants, further illustrates her concern with freedom of choice: “Though I admired the courage of the younger patients… I am not willing to go that route” (Yalom & Yalom, 2021, p. 41). She questioned the medicalisation of ageing and dying with a “one-size fits all” (p. 41) treatment approach, suggesting a critical awareness of how care systems can obscure individual agency. Perhaps most striking is Marilyn’s openness to reflect on her decisions regarding assisted suicide. In existential terms, this would represent the ultimate assertion of freedom: The right to shape not only one’s life but also one’s death. She wrote that she finds herself “grappling” (Yalom & Yalom, 2021, p. 77) with Nietzsche’s proposition that one should ‘die at the right time’, as it presents her with the basic choice about whether to preserve her life in the context of physical decline and suffering. Anticipating that her treatment options were becoming more limited, Marilyn asserted: “It seems to me that the decision to live or die should be primarily mine” (p. 77).

Marilyn’s reflections also reveal another end-of-life offshoot of the lifelong existential concern of freedom. This relates to the decreasing options available for self-description and how the imminence of their death may influence individuals’ view of themselves. In her opening chapter, she hinted at the significance of descriptive labels in this process: “I am trying to resign myself to the life of an invalid, or at least the life of a convalescent, as one referred politely to people like me in the past” (Yalom & Yalom, 2021, p. 20). A visit from friends who attended her literary salons and the outpouring of praise she received from these *salonnières* triggered careful introspection: “Could I really have been as kind and generous as my friends say I was?” (Yalom & Yalom, 2021, p. 90). She also described instances of emerging dissonance between her sense of self and her environment, such as standing in front of the newly emptied bookshelves in her study, despite her and Irving always having been “book people” (Yalom & Yalom, 2021, p. 127), and the changes imposed by her physical deterioration: “I can’t remember a time when I did not enjoy talking… But today, I am exhausted by lengthy conversation” (Yalom & Yalom, 2021, p. 20). A particularly powerful shift in the experience of the self is seen in her recollection of receiving treatment in the same hospital where one of her sons was born 50 years prior. She sharply contrasted the memory of his birth with her undertaking to, during his upcoming birthday celebrations, “do all I can not to look like a dying old lady…” (Yalom & Yalom, 2021, p. 74).

### Existential Concern 3: Isolation

In exploring Marilyn’s experience of existential isolation, it is important to appreciate the depths of her sense of connection. She often emphasised interconnectedness and her deep appreciation of meaningful relationships (Yalom & Yalom, 2021). As she contrasted her own experience with that of a fellow patient who faced treatment alone, she noted gratitude for not facing her suffering in complete isolation. She wrote: “I have become more aware of the extent to which my life is connected with the lives of others…” (Yalom & Yalom, 2021, p. 13), and added: “Ultimately, I have come to the understanding that one stays alive not only for oneself, but also for others. Though this insight may be self-evident, only now do I realize it fully” (p. 14). This suggests that proximity to death had deepened her sense of a relational ethic even further. Marilyn referred to numerous instances of close relationships being a source of comfort, as her loved ones often became loving caretakers (Yalom & Yalom, 2021). Her mention of gratitude for her continued ability to communicate after suffering a stroke further reveals the relational quality of what has provided comfort to her — visits with friends, shared laughter, the support of her children and her husband’s consistent caregiving. Her relationship with her husband, specifically, emerges as an emotional anchor: “But, most of all, it is Irv who sustains me…” (15). She described his caregiving and presence at all her treatment appointments (Yalom & Yalom, 2021) as not merely functionally supportive, but as an expression of enduring attachment and a testament to their shared life.

However, Marilyn also demonstrated insight into the unbridgeable gap between people that characterises the acceptance of existential isolation. In addition to the lingering sadness associated with this awareness (described under the first existential concern above), she wrote: “I hope I can at least live up to my expectations of dying in such a way as to cause as little pain to others… and to myself” (Yalom & Yalom, 2021, p. 103). Marylin also noted her gradual withdrawal of concerns over the well-being of her loved ones: “I have begun to detach myself from objects and people…I am trying to detach myself a little from the people I love most” (p. 78). As she carefully considered bequeathments of her most cherished possessions, she confronted the fundamental aloneness of experience: “…the stories attached to each one of our possessions will ultimately disappear” (Yalom & Yalom, 2021, p. 32). She also critically reflected on social expectations of generational custodianship over personally significant items: “Who will want all these objects? Just because they appeal to us and hold our memories does not mean that our children will desire them” (Yalom & Yalom, 2021, p. 30). She revisited this idea again, closer to her death:

“It is so hard to realize that all the books and papers and objects that have accompanied my life will have little meaning for my children and grandchildren. In fact, they will probably be a burden for them. I know I shall be doing them a service by getting rid of as much “stuff” as possible.” (p. 104)

A theme related to the end-of-life embodiment of this existential concern emerged, based on physical changes and the experience of pain, which seems to renew the focus on existential isolation. Marilyn wrote, “I am exhausted most of the time — as if…a foggy veil exists between me and the rest of the world” (Yalom & Yalom, 2021, p. 12). The differences between her and her husband’s readiness for her death also seem connected to physical changes. On one occasion, she disclosed that the toll of the treatments has become “too great a cost to stay alive” (Yalom & Yalom, 2021, p. 76) and asked: “How much longer must I live before I am allowed to die?” (p. 76). She then refuted his interjecting claim that she still enjoys herself sometimes by stating: “If I could place you inside my body for just a few minutes, you would understand” (p. 76). Confronted with the isolated experience of dying, Marilyn seemed to particularly appreciate the hospice care staff’s capacity to understand and support her physical needs (Yalom & Yalom, 2021).

### Existential Concern 4: Meaninglessness

Marilyn’s reflections on some of her most cherished memories and her proudest achievements highlight her lifelong striving towards a meaningful life. She noted having lived a “very satisfying long life” (Yalom & Yalom, 2021, p. 30), and reminisced often about her marriage, her family and her work. For example, when explaining her emotional ties to their home and its “liveable, lovable space” (Yalom & Yalom, 2021, p. 30), she stated: “How many birthday parties, book parties… and wedding receptions have we celebrated in the living room or outside on the back patio or on the front lawn?” (p. 30). She seemed to find continued meaning in her work. When referring to the manuscript on survivors of World War II, she noted: “I feel a special obligation to communicate these stories” (Yalom & Yalom, 2021, p. 130). Reflecting on the broad impact of her life’s work, she wrote: “It gives me a sense of pride to think that I, too, was part of the feminist movement that advanced women’s rights during the last two generations” (Yalom & Yalom, 2021, p. 101).

Rich with literary, spiritual, and philosophical allusions, she expressed a deeply human desire to have lived a life of value, noting the significance of the idea that one’s memory will endure (Yalom & Yalom, 2021). The theme of kindness that featured so prominently in her concern with personal autonomy also echoed throughout Marilyn’s reflections on her legacy and having lived a ‘good’ life, indicating this was also central to her meaning-making process. She recalled the inscription on a tombstone she encountered when writing about American burial traditions:

“‘To live in hearts we leave behind is not to die.’ To live in hearts…or, as Irv so often puts it, to ‘ripple’ into the lives of those who have known us personally or through our writing, or to follow the counsel of Saint Paul when he wrote: ‘though I have faith…, and have not charity, I am nothing’.” (Yalom & Yalom, 2021, p. 17)

Marilyn’s invocation of the dictum ‘The first is to be kind. The second is to be kind. And the third is to be kind’ was followed by her hope “to adhere to this dictum even as I anguish over my personal situation” (Yalom & Yalom, 202, p. 17). This reveals a renewed end-of-life tension between personal suffering and the enduring commitment to meaningful living. Therefore, pain represents a new theme of embodied experience of the existential crisis of meaninglessness. In her earliest reflections in the book, Marilyn asked, “…why should I want to live now with daily misery and despair?” (Yalom & Yalom, 2021, p.13). On the one hand, this suggests that having had the opportunity to create meaning in one’s life positively influences one’s acceptance of the imminence of death. On the other hand, it also suggests that prolonged physical suffering during the process of decline renews the crisis of meaninglessness. Throughout her reflections, the meaninglessness of her suffering underpinned her choices regarding a physician-assisted death. In the month before her death, Marilyn shared a form of meaning she created from her physical suffering by stating: “…In an odd way, I feel that I have ‘paid’ for any sins or wrongdoings I have committed during my lifetime” (Yalom & Yalom, 2021, p. 105).

### Conclusion and Recommendations

This single case application of Yalom’s existential psychology propositions demonstrated that all four existential concerns were present in Marilyn Yalom’s experiences of death and dying. Furthermore, the analysis has highlighted specific end-of-life concerns, which were interpreted as variations of the four existential themes specific to the context of imminent death. However, further research can explore whether they could alternatively support Binder’s (2022) proposition of a fifth existential concern, namely, *embodiment and emotional being*. Drawing from the work of Merleau-Ponty, Gendlin and Winnicott, Binder (2022) asserts that we are grounded by our awareness of our bodies, which allows us to experience feelings both about ourselves and outside events. Embodiment, which has not explicitly been included in Yalom’s framework, motivates the individual to balance feelings of vulnerability and receptivity with a sense of strength and agency (Binder, 2022). Research on the experience of embodiment, especially during the later phases of life, remains underexplored within research (van Rhyn et al., 2022). Marilyn’s specific end-of-life themes related to physical pain and suffering lend support to the need for further research to better understand embodiment as a potential existential concern in the context of failing health and advanced age.

Irvin Yalom’s synthesis of existential philosophy and psychotherapy has influenced contemporary therapeutic interventions and has resulted in these concerns becoming deeply embedded in clinical practice, as patients often grapple with questions of legacy, autonomy, connection, and purpose (Berman, 2019; Binder, 2022; Cunha et al., 2024). As such, the findings of this study readily result in potential practice recommendations to those who provide care for the terminally ill. Firstly, Marilyn’s gradual shift from an abstract acceptance of mortality to a personal confrontation with death’s imminence reveals a complex interplay of factors and demonstrates that the acceptance of death and dying is not a singular event, but a process influenced by emotional complexity, cultural expectations, philosophical and spiritual contemplation, and, significantly, attachment and separation. Clinicians are urged to be mindful of the distinction between acceptance of death and readiness to die. More specifically, support for the terminally ill should include the acknowledgement of continued fears about the physical process of dying, the desire for end-of-life reminiscence, as well as the lingering sadness over death as an impending final separation, even when someone has intellectually accepted the reality of their own decline.

Secondly, Marilyn’s confrontation of existential freedom in her last months involved candid reflections on personal agency, autonomy and responsibility and the drive to empower oneself to shape not only one’s life, but also one’s death. Care providers should be sensitive towards the significant agency that comes from choices about living and care arrangements and the bequeathment and management of cherished personal possessions. Furthermore, clinicians should demonstrate sensitivity to the impact of changing self-descriptors towards the end of life. To help protect the dying person’s sense of self, personal agency to live according to lifelong values should be supported. In this process, Marilyn’s reflections suggest that the identification of role models for “dying well” might be particularly impactful. Ultimately, these findings strongly support individualised medical care and continued advocacy for the autonomy provided by physician-assisted suicide, which remains an inaccessible care option to many.

Thirdly, Marilyn’s reflections also illuminate the importance of the relational core of human existence. Within the security of meaningful connections, the existential crisis of isolation may be reframed in the context of the solitary experiences of suffering and dying. Similarly, Marilyn’s reflections demonstrate how the experience of pain carries the potential to renew a confrontation with meaninglessness, even among those who are satisfied with the meaning they have created throughout their lives. In practice, mindfulness of the dying patient’s growing sense of isolation should also include acknowledgement of a gradual, intentional disconnection from loved ones as the fundamental isolation of the human experience moves to the foreground. Additionally, empathic and knowledgeable medical staff have the potential to provide a useful tether against overwhelming isolation and growing meaninglessness in a manner that loved ones cannot by openly discussing and actively managing the physical concerns and experiences during terminal illness.

## Data Availability

Data exclusively sampled from the public domain.
